# Supplementary material for: Antimicrobial susceptibility of western Canadian Brachyspira isolates: Development and standardization of an agar dilution susceptibility test method
Source: PLoS One. 2023 Jun 30;18(6):e0286594. doi: 10.1371/journal.pone.0286594 (PMC10313021; doi:10.1371/journal.pone.0286594)
Supplement: S1 Table — (DOCX) [file pone.0286594.s001.docx]

| ***B. pilosicoli*** | | ***B. hampsonii*** | | ***B. hyodysenteriae*** | |
| --- | --- | --- | --- | --- | --- |
| **OD_600_** | **CFU/ml** | **OD_600_** | **CFU/ml** | **OD_600_** | **CFU/ml** |
| 0.799 | 5.00 X 10^8^ | 1.016 | 3.57 X 10^8^ | 0.353 | 2.5 X 10^8^ |
| 0.425 | 3.67 X 10^8^ | 0.34 | 1.13 X 10^8^ | 0.186 | 1.2 X 10^8^ |
| 0.223 | 1.20 X 10^8^ | 0.097 | 2.65 X 10^7^ | 0.11 | 6.4 X 10^7^ |
| 0.131 | 7.67 X 10^7^ | 0.031 | 8.63 X 10^6^ | 0.062 | 3.4 X 10^7^ |
| 0.064 | 2.77 X 10^7^ |  |  | 0.039 | 1.5 X 10^7^ |
| 0.041 | 6.00 X 10^6^ |  |  | 0.037 | 1.6 X 10^7^ |
| 0.022 | 4.66 X 10^6^ |  |  |  |  |
