# Supplementary material for: Antimicrobial susceptibility of western Canadian Brachyspira isolates: Development and standardization of an agar dilution susceptibility test method
Source: PLoS One. 2023 Jun 30;18(6):e0286594. doi: 10.1371/journal.pone.0286594 (PMC10313021; doi:10.1371/journal.pone.0286594)
Supplement: Attachment — Submitted filename: Response to Reviewers .docx [file pone.0286594.s002.docx]

General comments:

Change in authors listed: In the initial submission, we had mistakenly included Dr. S.P. Gow in the manuscript file. Dr. Gow was not included as an author within the PLoS submission system and should not have been listed on the manuscript file. I apologize for this oversight.

The references have also been corrected as per the reviewer's suggestions.

Reviewer 1

Line 94 - A reference to the EUCAST breakpoint tables has been added as suggested.

Line 110 - Thank you for this comment, a description of this media has been added as suggested.

Lines 246-247 - Some modifications and additional information were added to this section of the paper to clarify. Antimicrobial free (positive control) plates were included with each batch of plates; growth was always observed on these plates. Had growth not been observed, the experiment would have been repeated. Regarding test endpoints, we defined the MIC as the lowest concentration where no hemolysis was observed; in this study we used hemolysis as an indicator of growth as opposed to a colony or turbidity which would be done for the majority of organisms encountered by the clinical diagnostic laboratory. Using hemolysis as an indicator of growth is an established method when working with *Brachyspira*. Finally, endpoints were only read by DGRS Kulathunga who performed these experiments during her PhD. Although, this isn't something that I can report in the paper, these observations were only made following a period of extensive training so the researcher was not a novice in the field when these experiments were conducted.

Lines 260-263, 435-441 and Table 3 - Thank you for this comment. We struggled with how best to present this data; to be fully transparent with our date we included a column describing the number of observations with MIC ≤ 0.25 µg/ml. A sentence was added just after line 260 to explain that repeated observations ≤ 0.25 µg/ml were considered reproducible. In the discussion section lines 649-652 a sentence was added to address the limitation in interpreting reproducibility for isolates with MICs ≤ 0.25 µg/ml.

Discussion - Thank you for raising this point. We have added a recommendation for how we might see this method implemented into a clinical diagnostic laboratory on lines 653-658.

Reviewer 2:

Thank you for the comment recognizing the volume of work reflected in this manuscript. We agree that there is a lot of data here and that this manuscript includes 2 large bodies of work (1. method development and 2. testing isolate collection). We had lengthy discussions on whether to lump these data together and struggled with what would be the best approach, in the end we felt that for the benefit of the *Brachyspira* literature it would be better to present this data as a single body of work. We felt that this manuscript was much stronger if both parts were combined; the length of the paper and the number of figures we wanted to include factored into our decision to submit to PLoS One.

Point 1 - We have added a description of what test factors are standardized according to the CLSI and EUCAST on lines 95-98.

Point 2 - Thank you for these detailed comments.

- We have reviewed which CLSI documents are cited throughout the manuscript and made changed which documents are cited where necessary.
- Regarding line 234 - you are correct, the CLSI does not recommend TSA. This sentence has been modified to emphasize that the media was prepared according to the CLSI guidelines (procedure for measuring antimicrobial powders etc.) but that the antimicrobials were then incorporated into TSA vs. Mueller-Hinton agar.
- We have also modified the CLSI reference at line 234 - this should not in fact be M100 but the M07 document which described the procedure for agar dilution testing.

Point 3 - We absolutely appreciate that the method described here is somewhat complicated, and as an agar dilution protocol it is certainly more laborious than other methods such as disc diffusion or commercially prepared broth microdilution. However, we believe that we have demonstrated that the current method, unlike others previously reported, is sufficiently reliable to be considered as a tool in clinical diagnostic laboratories. We fully accept your criticism of 'what to do with the data', we are not yet at a point where we have validated clinical breakpoints as we do with other veterinary pathogens, so caution will be required when interpreting this data. This study is a necessary first step, before interpretive criteria can be developed, reliable and standardized test methodologies need to be developed. Ultimately, high quality antimicrobial susceptibility data is a cornerstone of diagnostic bacteriology; it is required to guide therapeutic selection, promote antimicrobial stewardship to reduce the selection pressure for resistance and finally to detect the emergence of antimicrobial resistance.

With respect to stratifying data by species group, this was done for two primary reasons. First, we wanted to ensure that the most granular data possible was presented to allow researchers to re-interpret our data in the future. Second, because differences in intrinsic resistance between species are well recognized (see EUCAST Expected Resistant Phenotypes v1.2 January 2023 table for examples), we wanted to ensure that any such phenomenon was not masked by aggregating data within a single group.

Point 4

- Figure 5 - A figure legend has been added to explain this figure.
- Table 4 - A sentence has been added (lines 677-680). The main reason that it is important to document that the isolates in this study were from epidemiologically distinct production systems is to be transparent about how biased (homogeneous) our isolate collection is. If all isolates originated from the same farm it would not have been reasonable to discuss MIC distributions because the isolates would have been subjected to the same antimicrobial selection pressures.

Point 5

- Line 652-653 - A clarification has been added here. We want to highlight the fact that while methodological differences preclude comparisons between labs, results generated over time from within a single lab may be compared with less uncertainty.
- Line 668-669 - We have decided to delete this sentence in accordance with your suggestion.

Reviewer 3:

Point 1

- These three isolates were selected because they represent the three most commonly identified *Brachyspira* species causing infections in pigs in our region. Clarification was added to explain that these isolates were selected for this reason.

Point 2

- In this study we did not include isolates posessing particular resistance genes or resistance conferring mutations. Because the isolate collection we tested was a set of diagnostic isolates from our institution, this information was not available. Before doing this study we were in the difficult position of having neither genotypic resistance data nor phenotypic susceptibility results for our collection which is why we performed our assays in triplicate and took other measures as described to be confident with our results. However, now that these isolates have been characterized, screening for genetic mutations is an obvious next step, although we believe that this is beyond the scope of this investigation.

Point 3

- The data relating OD to CFU/ml is now included as "S1 Table".

Point 4

- Thank you for this suggestion, this has been clarified in the manuscript.

Point 5

- This is work which was previously done in our lab, this data is now provided as supplementary materials as suggested in point 3.

Point 6

- When working with *Brachyspira* it is widely reported that broth cultures are incubated at a lower temperature than agar cultures, I do not have an explanation for why this is required/is common practice. I would speculate that incubating plates at 42C serves to make primary cultures more selective (inhibiting organisms which don't grow at 42C), once a pure culture is obtained then lower temperatures (39C or some in the field also use 37C) can be used for broth cultures.

Point 7

- The sentence has been clarified, we did inspect broth under the microscope for the presence of motile spirochetes.

Point 8

- These dilution ranges were selected to ensure that we assessed the optical density of a wide range of concentrations. In our experience, *Brachyspira* species sometimes do not grow to a sufficiently high density and so we wanted to ensure that we were able to capture dilutions less than 1:2. It was critical to have multiple data points which fell within the linear portion of the curve relating OD to concentration in order to define the equation describing this relationship.
- With respect to the minimum inoculum, we anticipated that the minimum inoculum required to start a culture would be much less than would be required for detection in a spectrophotometer. Indeed, on agar we were able to observe growth with very low CFU counts.

Point 9

- The two media which were used in this study were utilized for different phases of the investigation.
- BJ agar is a selective, antimicrobial containing media (colistin, vancomycin, spectinomycin, spiramycin and rifampin) which is used for primary isolation from clinical samples. We also use this media routinely when working with cultures of *Brachyspira*. A description of
- TSA agar was used as a base for susceptibility testing, it was important to use a media which did not contain antibiotics for this application.
- A description of the antibiotics contained within BJ agar has been added as per the recommendation of Reviewer 1.

Point 10

- We have added a description of the way the phylogenetic tree was constructed where you suggested.

Point 11

- For this phylogenetic tree we would like to suggest that we keep the data for the same isolates which were initially presented. The ATCC strains of *B. hyodysenteriae, B. pilosicoli, B. innocens* and *B. murdochii* were included because these are the type strains of each species; because the purpose of this comparison was to help identify the species of our study isolates we only wanted to compare to the reference strains for each. With respect to *B. hampsonii* 30599 and *B. hampsonii* 30446 - these reflect the two *B. hampsonii* genomevars, the figure legend has been updated to clarify the identity of these isolates.

Point 12

- Clarifying language has been added to the end of the section describing sequencing of the *nox* gene.

Point 13

- In susceptibility testing it is critical to ensure that a consistent, standardized inoculum is tested. Adjusting all broths to a the density reported was therefore critical to the development of the standardized method we report.

Point 14

- Thank you for this comment. The decision to use a single equation was a matter of practicality; in developing a test that we hope can be applied in a diagnostic laboratory setting it would be infeasible to have separate criteria for each species. While this does introduce a level of variability into the protocol, this is accounted for by the target range of CFU/ml (1-2 × 10^8^ CFU/ml). This range of acceptable concentrations is recommended by the Clinical and Laboratory Standards Institute and allows for a single density of organisms (MacFarland 0.5) to be used in susceptibility testing across diverse clinical isolates (Gram-positives, negatives and anaerobes).

Points 15 and 16

- The differences in maximum OD reported for each species reflect the linear portion of the standard curve over which the equation to relate OD:CFU/ml was derived. At an OD >1 there was not a linear relationship between OD/CFU/ml.

Point 17

- In this investigation we did not study the growth kinetics of *Brachyspira,* all of the measurements of culture density which were recorded were endpoints and collected for the purpose of calculating CFU/ml.

Point 18

- Prior to performing any work with broth cultures they were assessed microscopically. While we did not specifically look for clumping/aggregation, this not a phenomenon that we have previously recognized. When cultures were examined prior to measuring OD they were always fresh cultures which were motile.

Point 19

- No, as per our response to point 2 we did not assess this culture collection for the presence of resistance genes or resistance conferring mutations. While this is an obvious next step, it was beyond the scope of this study.

Point 20

- Thank you very much for raising this point. This was an oversight on our part, the sequences have been submitted to GenBank and BankIt accession numbers incorporated into the text.
